# Baltimore College of Dental Surgery, and American Dentists

**Published:** 1851-07

**Authors:** 


*Baltimore College of Dental Surgery, and American Dentists.*—This Institution appears to be in a flourishing condition, and its influence on the character of Dental Science, and the Dental profession in this country can not but be salutary. We learn from our esteemed contemporary, 'the American Journal of Dental Science,' that, at the last annual commencement, the degree of Doctor in Dental Surgery was conferred on nine graduates. The addresses on the occasion, by Charles G. Davis, on behalf of the graduating class, and by Dr. Eleazor Parmly, Provost of the college, are given in the Journal just mentioned, and are both able and interesting.

The Candidates for graduation in this Institution, before being admitted to a degree, are examined by a joint Committee composed of distinguished members of the Medical and Dental professions. This course was adopted in accordance with the recommendation of the American Medical Association.

C. W. Harvey, M. D., Surgeon Dentist, of this city, has been appointed a member of the Examining Committee for the next collegiate year. This is a merited tribute to the high professional standing of Dr. H. who, as will be perceived by another article in this Journal, has recently been elected president of the Buffalo City Medical Association.

We believe it is generally conceded that the science and art of Dentistry have been brought to greater perfection in the United States than in any other country. Several American Dentists have established themselves on the other side of the Atlantic and hold prominent positions. In a late number of the Boston Medical and Surgical Journal, the Editor, who writes from London, says, that on visiting the industrial exhibition, and "looking over the specimens of American Dentistry, I came to the conclusion, as I have done frequently before, that Europe cannot match the best workmanship of our country. By contrasting the specimens of different countries, it is no difficult problem to determine who are the best workmen."

